# Comparative Evaluation of Microleakage in Class II Restorations Using Open- and Closed-Sandwich Techniques With Zirconomer as an Intermediate Material: An In-Vitro Study

**DOI:** 10.7759/cureus.30109

**Published:** 2022-10-09

**Authors:** Prachi Taori, Pradnya Nikhade, Manoj Chandak, Joyeeta Mahapatra

**Affiliations:** 1 Department of Conservative Dentistry and Endodontics, Sharad Pawar Dental College, Datta Meghe Institute of Medical Sciences, Wardha, IND

**Keywords:** composite, zirconomer, close sandwich technique, open sandwich technique, class ii cavity, microleakage

## Abstract

Aim

To evaluate and compare the microleakage In class II restorations using open- and closed-sandwich techniques with zirconomer as an intermediate material.

Material and method

Twenty-six non-carious mandibular first molars were selected and randomly divided into two groups (groups 1 and 2 where n=13). A standardized Class II preparation was made with the cervical margin 1 mm below the cementum-enamel junction. Samples of group 1 were restored using the open-sandwich technique and samples of group 2 with the close-sandwich technique, and zirconomer was used as an intermediate restorative material. Following that, the restorations underwent 200 heat cycles with dwell times of 20 seconds at 5°C and 55°C. Dye penetration and scanning electron microscope (SEM) analysis using the replica approach were used to assess adaptation at the cervical margin. The data were statistically analyzed using the Paired T-test (*p*<0.05).

Results

Lower dye penetration was seen in the open-sandwich technique compared to the closed-sandwich technique (*p*<0.001).

Conclusion

When comparing the open-sandwich technique with the closed one, it was observed that less microleakage was seen in the open-sandwich technique as it has better marginal adaptation and fewer voids.

## Introduction

The primary aim of dentistry is to have material with a minimum toxic effect on pulp and an acceptable seal at the microscopic level when used for the restoration of a cavity [1]. The material used in restorative dentistry should be biocompatible, with a coefficient of thermal expansion similar to that of the tooth, better marginal sealing, chemical bond with tooth structure, and better colour stability [2]. Considering the cause of failure of restorative material, microleakage is the most common cause as it leads to secondary caries and irritation to the pulp. Microleakage in conservative dentistry is the foremost setback encountered in clinical practice leading to compromised restorations [3]. Kidd in 1976, was the first to give a definition of the term microleakage and its clinical aspects. “It may be defined as the clinically untraceable opening for bacteria, fluids, molecules or ions between a cavity wall and the applied restorative material to it” [2]. Microleakage can lead to secondary caries, hypersensitivity, reduced life of restoration, and marginal discolouration. The main area of concern is to have a material that bonds effectively with the walls of the prepared cavity [4].

For decades, amalgam has been used successfully for restoration of class II cavities but as the material is not aesthetic and also there are hazardous effects of mercury, it is being replaced by composites [5]. Composite resin is the most commonly used tooth-coloured restorative material. But difficulties are still there when it is used directly in proximal restoration. Among all the difficulties, the most common is polymerization shrinkage which leads to the formation of a gap [6]. Microleakage in class II restoration mostly occurs due to masticatory stresses and thermal changes which leads to secondary or recurrent caries. Few clinical studies have shown that it is the most obvious reason for the replacement of restoration [7]. The bond between resin and enamel is generally satisfactory but the bond with dentin is not adequate, which leads to the formation of a v-shaped defect at the cavity margin situated in the cervical region due to less enamel during polymerisation [8].

To overcome this problem of marginal leakage, various clinical techniques have been introduced. McLean and Wilson introduced a technique in 1977 in which a glass ionomer cement base was used to substantially replace the composite resin restorative material in the proximal box [9]. This technique is known as "composite-laminated GIC" or "sandwich" restoration [10]. Earlier the filler that was used in the sandwich technique was glass ionomer cement. But this material undergoes acid-base reaction and also it requires time to set. So, instead of using conventional glass ionomer cement, zirconomer (zirconia + GIC) is being used. This material has adequate strength and durability and when compared to amalgam, the hazardous effect of mercury is eliminated [5].

In the traditional “closed-sandwich” technique, zirconomer is placed in the basal area of the proximal box not reaching the external cavo-surface. following the setting of the zirconomer cement, it is etched using 37% phosphoric acid for 20 seconds. Then dentin bonding agent is applied and light cured for 20 seconds. Finally, the composite resin is placed first in the proximal box and then on the occlusal surface. In this technique, zirconomer is not exposed to the oral environment.

 In the “open-sandwich” technique, zirconomer is placed in the basal area of the proximal box up to the external cavo-surface and composite resin restoration is done to the same level. The primary advantage of the "open-sandwich" technique is that it provides a larger surface area of zirconomer which in turn allows the buffering of the acidic pH of the oral cavity. So this investigation was designed to evaluate microleakage in class II restorations using open- and closed-sandwich techniques using zirconomer as an intermediate material.

## Materials and methods

Data collection 

Institutional ethical committee grants permission to conduct this study (Ref No. DMIMS(DU)/IEC/2020-21/134). Informed consent was taken from the patients, whose teeth were removed due to periodontal problems, to use their extracted teeth for the study. Human permanent mandibular molars extracted due to loss of periodontal support were collected with the help of the Department of Oral and Maxillofacial Surgery, Sharad Pawar Dental College, Sawangi (M), Wardha. Among these, 26 sound healthy teeth were collected.

Inclusion criteria

Teeth with an absence of caries, restoration, cracks, and white spots were selected.

Cavity preparation

**Figure 1:**
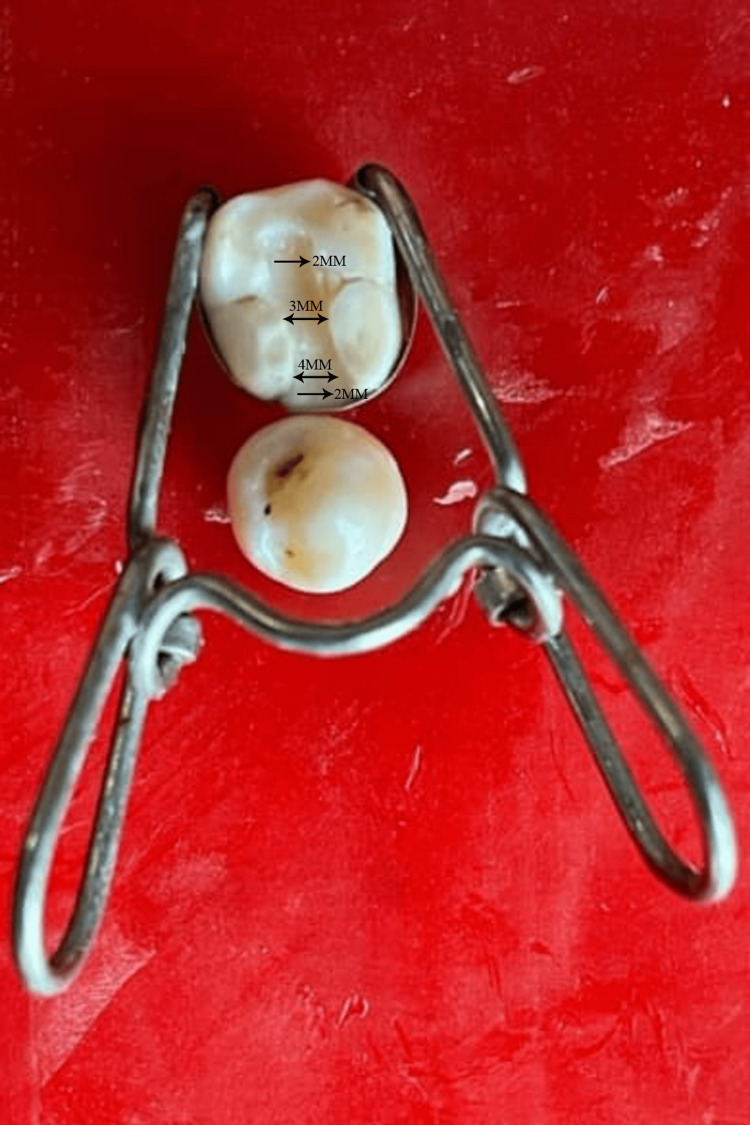
Figure showing dimensions of prepared class II cavity

All samples were cleaned and kept in distilled water. A mesio-occlusal class II cavity was prepared using a round bur (Mani diamond rotary instrument, Tochigi, Japan) on all the teeth. the dimensions of the cavity on the occlusal surface were such that it was 3 mm wide and its depth was 2 mm. In the proximal box, the cervical margin was placed 1 mm below the cemento-enamel junction. The width of the proximal box was 4 mm bucco-lingually and the depth was 2 mm as shown in Figure 1. These dimensions were then checked using Boley's gauge (Trust and Care, New Delhi, India). A ±0.3 mm tolerance in the measurements was considered acceptable for including the specimen in the trial. None of the margins of the preparation had a bevel. After every fifth cavity preparation, new bur was used. Cavity preparation for each tooth was done by the same person so as to standardize and avoid bias in the result of the study. 

Restorative procedure

Random division of the teeth was done into two groups of 13 specimens each according to the technique employed for restoration. Group 1 comprised of open-sandwich technique and group 2 comprised of close-sandwich technique.

In Group 1 (open-sandwich technique), air-drying of the cavity was done and a 1-mm thick base of zirconomer (Zirconomer, Shofu, Japan) was placed at the cervical margin up to the external cavo-surface and it was allowed to set. As soon as the zirconomer was set, it was etched using 37% phosphoric acid for 20 seconds. Following this, a dentin bonding agent was applied and light cured for 20 seconds. Then, composite resin (Dentsply Spectrum Composite kit) was placed in a thickness of 2 mm over the gingival wall of the proximal box. The composite was then packed inter-proximally towards the metal matrix. The restoration was done up to the occlusal surface strictly following the inner surface of the matrix band. After the adaptation of the material, it was light-cured. Then horizontal increments of 2 mm of thickness were placed at the occlusal margin of the cavity leaving the zirconomer exposed to the oral environment.

In Group 2 (closed-sandwich technique) after placement of the zirconomer at the base of the proximal box such that it falls just short i.e., 1.5 mm of the external cavo surface, etching, and bonding of the zirconomer were done. Composite resin was placed in the proximal box and occlusal surface, leaving the zirconomer encased within the preparation.

Dye penetration test

**Figure 2:**
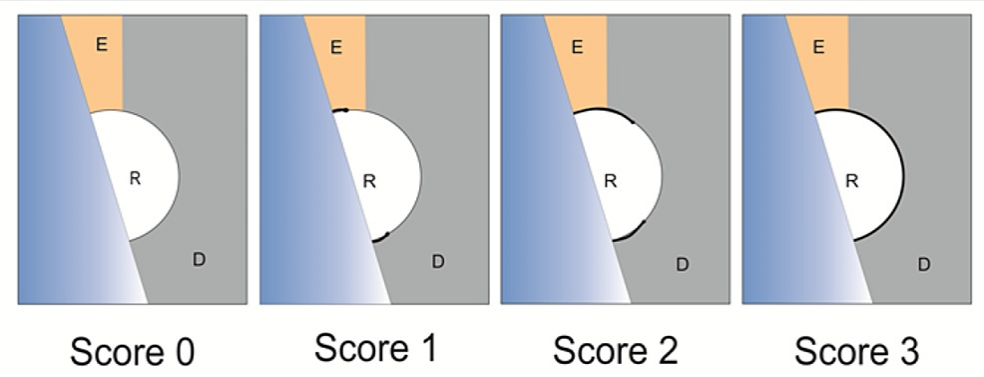
Scoring system to measure dye penetration E-Enamel; D-Dentin; R-Root surface

Samples were then processed for thermocycling. The temperature of the bath was kept at 50° to 55°C for 200 thermocycles. The cycle in each bath lasted for 30 seconds with 10 seconds of transfer time. With the help of modelling wax, the apex of the root was sealed to prevent the ingress of dye. The samples were then coated with nail varnish, applying two coats around all surfaces of teeth leaving 2 mm of the marginal area around the restorations. Immersion of the sample was then done in methylene blue dye, keeping it in the dye for one day at a room temperature of 37 degrees. Samples were then washed under running water for one minute and then air dried. In the next step, restorations were sectioned longitudinally through the centre of the tooth. They were sectioned by using a diamond disc (Taboom Diamond Disc DDOOO1 4A HP) along with water as coolant. Sections were examined at 32X magnification under a stereomicroscope (Lobovision, India). The samples were then scored according to the scoring system given by Pontes DG, 2002 as scores of 1, 2, and 3 (Figure 2). The scores were evaluated by two examiners who were precalibrated to measure the dye penetrated. The samples were coded and mixed for blinding so that the investigators could not identify the group of samples.

Statistical analysis

All data were statistically analysed using SPSS software, version 15.0 (SPSS Inc., Chicago) using a parametric paired T-test. Statistical significance was taken as a p-value less than 0.05.

## Results

In light of the current study, both the tested groups showed some amount of dye penetration with more dye penetration seen in the closed-sandwich group. Table 1 reports the values of microleakage scores recorded at the cervical margin in the two experimental groups, along with the statistical significance of the between-group difference. Paired T-test showed that lower microleakage was seen in the open-sandwich technique than in the closed-sandwich technique.

**Table 1:** Descriptive values of microleakage for different techniques

	Open sandwich technique	Closed sandwich technique
Mean	0.53	0.76
Standard Deviation	0.51	0.59
Standard Error	0.14	0.16
Minimum	0	0
Maximum	1	2

Table 2 depicts a comparison of microleakage between the open-sandwich technique and the closed-sandwich technique. Scanning electron microscopy (SEM) observations at the gingival margins of Group 1 specimens revealed adequate marginal adaptation without interfacial gaps or voids. It was observed that a slight overhang was seen in one of the specimens, probably due to an inaccurate matrix placement whereas marginal adaptation was less satisfactory in Group 2 specimens. Thus, it can be interpreted that marginal adaptation is better in the open-sandwich technique leading to lesser microleakage when compared with the closed-sandwich technique.

**Table 2:** Comparison of microleakage between open-sandwich technique and closed-sandwich technique

Parameters	Open sandwich technique)	Closed sandwich technique	Difference	p-value
Shear Bond Strength	0.53 ± 0.51	0.76 ± 0.59	0.23 ± 0.59	0.19

The null hypothesis is rejected from the results which showed there was a significant difference between open- and closed-sandwich techniques with zirconomer as an intermediate layer in class II restoration that extended below the cemento-enamel junction. Thus it can be said that the closed-sandwich technique does not provide good marginal sealing at the cervical margin of the restoration.

## Discussion

The quest for a dental cement having the best adhesion to enamel and dentine of tooth so as to seal the interface between restoration and tooth without compromising mechanical and physical properties is never-ending [11]. Microleakage by meaning is the passage of small amounts of fluids along with debris in the gap between a dental cement and the cavity margin at the surface. Attributable to ill-fitting margins of cement or restoration, microleakage cannot be measured clinically. Recurrent caries and pulpal inflammation are the most common sequelae of leakage apart from sensitivity and discolouration and compromised aesthetics of tooth-coloured restorations. Marginal leakage is an important parameter from a clinical view as it can affect the longevity of the restoration and if compromised, leads to its failure [12].

At present, there are various methods to measure microleakage (Alani and Toh, 1997) [13]. Some newer advancements with microleakage testing are radioisotopes, dyes, air pressure, neutron activation analysis, pH changes, and scanning electron microscopy [14]. Though traditional and one of the oldest methods, dye penetration is one of the most common methods employed to check leakage. Its advantages include less technique sensitivity, ease of performance, and cost-effectiveness (Raskin et al., 2001) [15]. In the present study, 2% methylene blue dye was used to test the samples. The important property of methylene blue is its small molecular weight which is smaller than some bacteria found in the oral habitat and also it can penetrate the dentinal tubules easily [16].

Thermocycling is a technique commonly used for in-vitro studies to test the predictability of dental cement in conditions that mimic intraoral temperature changes by exposing the restorations to temperature changes that can occur within the oral cavity [17]. The degree of dye penetration is not influenced by the number of thermal cycles. Therefore, in the present study, the samples were exposed to 100 cycles for 30 seconds [18].

In this in-vitro study, two different restorative techniques for class II restoration were evaluated to measure their sealing performance. Here the null hypothesis was rejected, as the dye penetration was least in the open-sandwich technique when compared with the close-sandwich technique. Korkmaz et al. in their study demonstrated that the closed-sandwich technique requires greater operator skill and achieves relatively poor marginal adaptation [19]. In various microleakage studies, it was found that the dye tracer penetration was greater when the margins were located in dentin than in enamel [20,21]. This may be attributed to the fact that the contraction forces which occur within the polymerizing composite resin are sufficient to disrupt the bond between the zirconomer and dentin, mainly in the initial stages of cement maturation [22].

In the present study, the sandwich technique with zirconomer as the liner was used as it reduces the bulk of composite resin in restoration. According to the literature, the polymerization shrinkage stresses were relieved by 20-50% and volumetric contraction by 41% [23]. Other characteristics were also noted to have improved, including fluoride release (found in the original glass ionomers), true adhesion, reduced microleakage, low water solubility coefficient and fluid absorption, longer working times, increased fracture toughness, and better mechanical and chemical characteristics.

From the statistical analysis, it was found that there was no significant difference in microleakage at the gingival and occlusal area in the open sandwich technique. This was in accordance with Khadim AJ [24] and Pouyanfar et al. [25] studies. This is explained by enamel's relatively high mineral content, consistent structural formulation, and ability to serve as a trustworthy substrate for micromechanical bonds to composites. The most difficult substrate for bonding is gingival dentin. Since the lining material used in the sandwich technique is sufficiently flexible to resist the stress caused by polymerization shrinkage and can also favourably dissipate stress caused by thermal variations, water absorption, and occlusal load across the interface, it serves as an "elastic buffer" [26].

In contrast to the open-sandwich technique, the microleakage was significantly higher in the gingival area than in the occlusal area in the closed-sandwich technique. This was in accordance with Nematollahiet et al. [27]. This may be due to polymerization shrinkage and a high coefficient of thermal expansion which leads to changes in dimensions [25].

The purpose of this study was satisfied as it was seen that microleakage was less in the open sandwich technique when compared with the close-sandwich technique. These in-vitro microleakage studies are the basis for any in-vivo studies as they provide initial information to overcome negative results to compare different new restorative materials and techniques.

This in-vitro study's limitations include the lack of stimulation of the dynamic intraoral thermal changes brought on by regular eating and drinking, the absence of outward flow of the dentinal fluid, completely altered dentinal surface by extraction, and only ideal cavities being prepared. As a result, there is a poor correlation between in-vivo and in-vitro conditions. However, these in-vitro microleakage studies serve as the foundation for all in-vivo studies since they offer initial data that may be used to overcome discouraging findings and contrast various novel restorative materials and methods.

## Conclusions

In accordance with the suggested technique and within the constraints of an in-vitro study, it can be concluded that none of the techniques tested in the study was free of microleakage, and cavities that were filled with the open-sandwich technique showed less microleakage with better marginal adaptation and fewer voids than the closed-sandwich technique. Zirconomer placed in the gingival floor of class II composite restoration (open-sandwich technique) may be a practical method to reduce microleakage.
